# The Nose and the Lung: United Airway Disease?

**DOI:** 10.3389/fped.2017.00044

**Published:** 2017-03-03

**Authors:** Amelia Licari, Riccardo Castagnoli, Chiara Francesca Denicolò, Linda Rossini, Alessia Marseglia, Gian Luigi Marseglia

**Affiliations:** ^1^Department of Pediatrics, Fondazione IRCCS Policlinico San Matteo, University of Pavia, Pavia, Italy

**Keywords:** allergic rhinitis, asthma, non-allergic rhinitis, local allergic rhinitis, airway disease

## Abstract

Epidemiologic, pathophysiologic, and clinical evidences recently revealed the link between upper and lower airways, changing the global pathogenic view of respiratory allergy. The aim of this review is to highlight the strong interaction between the upper and lower respiratory tract diseases, in particular allergic rhinitis and asthma.

## Introduction

During the second century, Claudius Galenus identified the effect of the upper airway on the lower airway and defined the nose as a “respiratory instrument” in his work “De usu partium.” Nevertheless, the concept of the upper and lower respiratory passages being a continuum and forming a single unified airway has been highlighted only over the last 10–15 years, starting from the Allergic Rhinitis and its Impact on Asthma (ARIA) World Health Organization workshop (1–3).

The mechanisms of nose and lung interaction are complex, not entirely understood, and they have been long investigated (4). Dating back to 1919, Sluder hypothesized the existence of a nasal-bronchial reflex (5), supported by the evidence of a similar innervation of both upper and lower airways (6). More recent studies have demonstrated the role of localized inflammatory changes in the upper and lower airways, which lead to a systemic response (7–9).

The aim of this review is to underline the strong anatomical, epidemiologic, pathophysiologic, clinical, and therapeutic evidences (summarized in Table 1) supporting the connection existing between the so-called United Airway Disease (UAD). We focus our attention on rhinitis and asthma, the most frequent and chronic inflammatory diseases of the upper and lower airways, presenting with several phenotypes.

**Table 1:** **The evidence and the mechanisms of nose and lung interaction**.

Anatomical and histological evidence	Epidemiologic evidence	Pathophysiologic evidence	Emerging biomarkers	Clinical and treatment evidence
The nasal and bronchial mucosae consist of ciliary epithelium resting on a basement membrane. Beneath the basement membrane is the lamina propria, glands, and goblet cells (17, 18)	19–38% of patients with allergic rhinitis (AR) have concomitant asthma and 30–80% of asthmatics have AR (15, 19)	The communication between the upper and lower airways is suggested to be *via* a bone marrow- derived systemic inflammatory response (17)	The role of microbiome: children being raised on traditional farms have a much lower prevalence of allergic disease as children grown up in urban settings (15, 18)	The treatment of AR can improve asthma symptoms (15, 18)

Both act as a transport system moving air in and out of the lungs (16)		The presence of epithelial basement membrane thickening, the typical hallmark of lower airway remodeling, not only in asthmatic patients but also in atopic patients without asthma and patients with AR (24)	The role of microRNA (miRNA): presence of the same particular miRNAs in different pathogenetic mechanisms of both AR and asthma, such as IL-13 pathway, GATA binding protein 3, and mucin secretion (42)	Decrease in asthma symptoms and AR after intranasal corticosteroid treatment of rhinitis (41)

Both provide defense against inhaled foreign substances, with most particles of 5–10 μm diameter filtered out by the nose, and irritant and soluble gases being extensively removed by dissolution in nasal secretions. The lower airway functions similarly, with smaller inhaled particles that reach the lower airway being trapped and cleared by the mucociliary escalator (19)		In non-allergic asthma it has been highlighted the importance of the presence of IgE in the bronchial mucosa, as in the nasal mucosa in local allergic rhinitis (10)		Leukotriene receptor antagonists are known to be useful for long-term management of asthma patients complicated by AR (42)

				The recombinant, humanized, monoclonal anti-IgE antibody Omalizumab improved nasal and bronchial symptoms and reduced unscheduled visits due to asthma (45)
				Allergen immunotherapy is effective for treating both rhinitis and asthma (38)

## Rhinitis and Asthma

The term rhinitis includes several different phenotypes and the diagnosis of each of these subtypes is often an interesting challenge (10). It is usual to divide rhinitis in allergic rhinitis (AR) and non-allergic rhinitis (NAR), based on allergological evaluation (10, 11). AR is a disease of the nasal mucous membranes, induced by an IgE-dependent inflammation after the exposure to allergens (12). Symptoms of AR include rhinorrhea, nasal obstruction or blockage, nasal itching, sneezing, and postnasal drip, which reverse spontaneously or after treatment. AR is classified based on the frequency of symptoms in intermittent (symptoms occurring on less than 4 days per week or for less than 4 weeks per year) or persistent (symptoms occurring on at least 4 days per week or for more than 4 weeks per year). In addition, considering the severity of symptoms, AR can be divided into mild (normal sleep; no impairment of daily activities, no troublesome symptoms) or moderate to severe (one or more of: abnormal sleep; impairment of daily activities, and severe symptoms) (12). On the other hand, NAR includes a group of diseases characterized by the presence of at least two nasal symptoms, such as pruritus, rhinorrea, obstruction, sneezing in patients who are not sensitized to any allergen. The diagnosis is mainly based on exclusion (11). In the last few years, a new phenotype called local allergic rhinitis (LAR) has been defined (13). It is characterized by symptoms suggestive of AR due to a localized allergic response in the nasal mucosa, in the absence of atopy assessed by conventional diagnostic tests. The immunological characteristics of LAR are nasal Th2 allergic inflammation, with positive response to nasal allergen provocation test, and nasal production of IgE and inflammatory mediators (14). However, a better understanding of the underlying immune mechanisms of this disease is essential for developing diagnostic methods and targeted therapies.

According to the recent GINA guidelines, asthma is a chronic disease, potentially serious, characterized by a reversible airway obstruction, chronic airway inflammation, and bronchial hyper-reactivity (15). It is diagnosed by the pattern of respiratory symptoms, such as wheeze, chest tightness, dry cough, and shortness of breath, that vary over time and in intensity, together with variable expiratory airflow limitation, induced by the bronchoconstriction, and morphological changes in the bronchial wall (“remodeling”). Symptoms are usually worse during the night or upon awakening, and the principal triggers are viral infections, allergens, tobacco smoke, exercise, and stress (15).

## Anatomical Evidence

**Figure 1:**
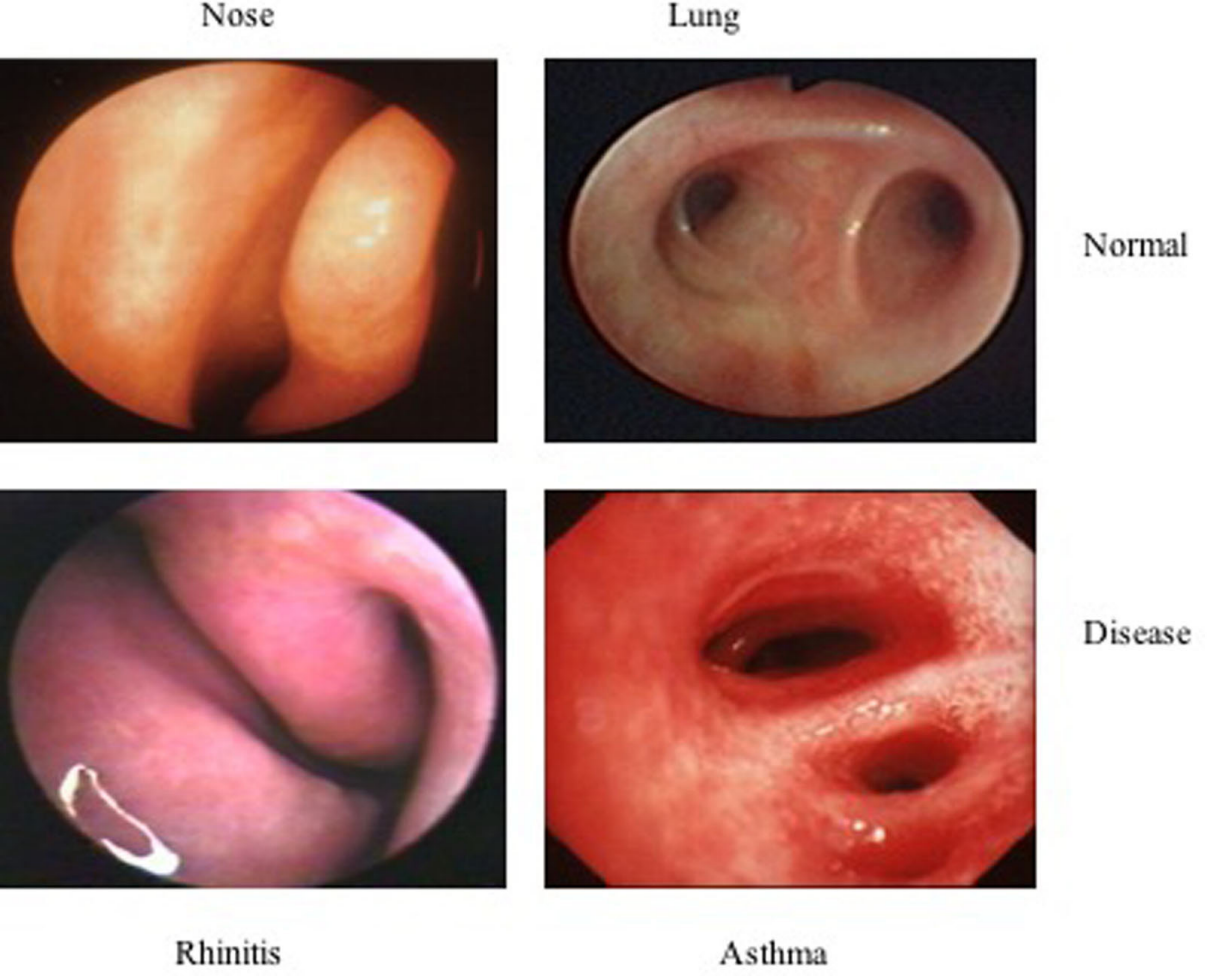
**Macroscopic pathological characteristics**.

**Figure 2:**
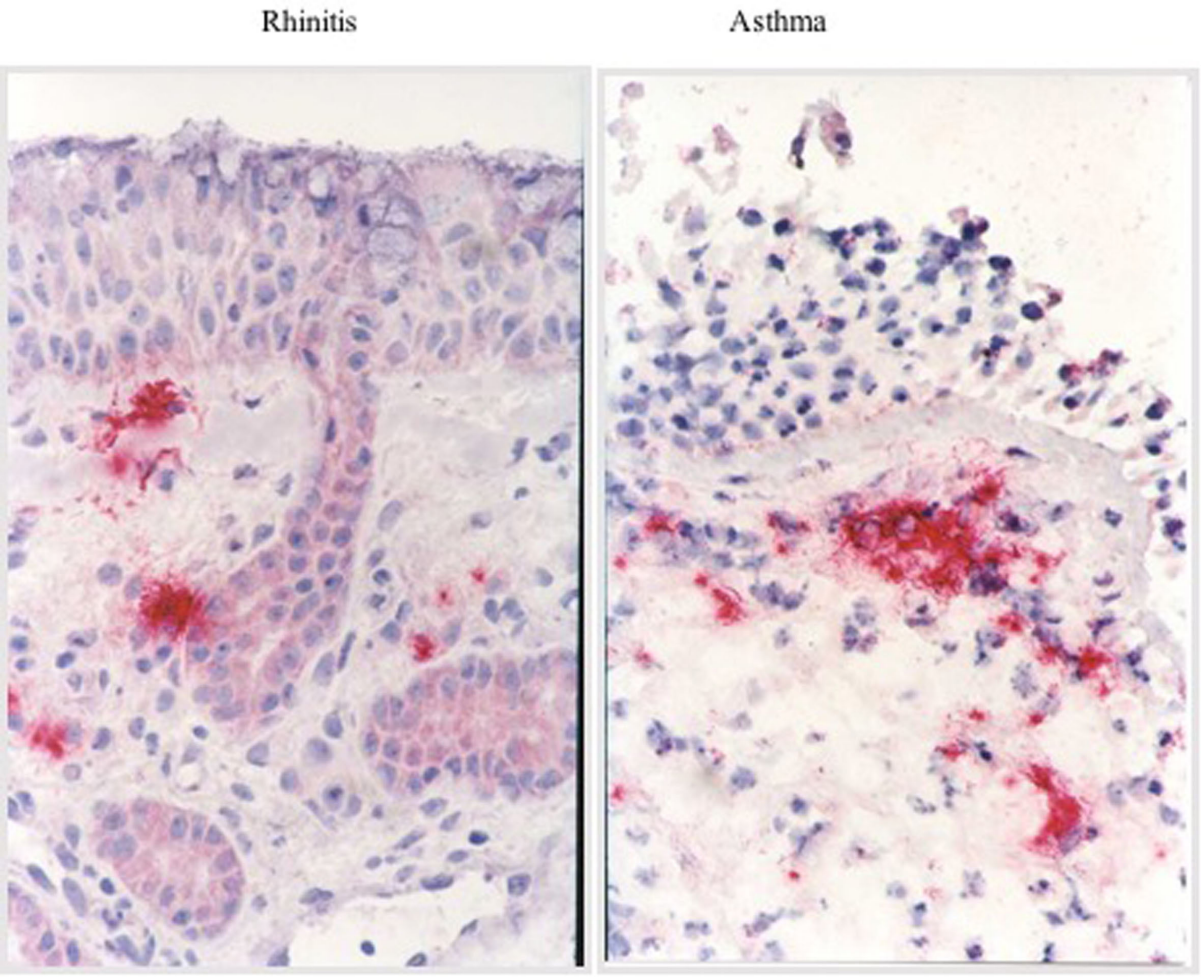
**Histological appearance**.

Anatomically, the respiratory tree is divided into upper (nose, pharynx, and larynx) and lower respiratory tract (trachea, bronchi, bronchioles, alveolar duct, and alveoli), separated by the larynx. Functionally, it is divided between conductive airways and gas-exchange region of the lungs, consisting of the respiratory bronchioles with the air cells (16). Although in daily practice, the nose and the lungs are considered as separate entities and treated by two different specialists, the upper and the lower respiratory tracts have anatomical and histological similarities, including the basement membrane, lamina propria, ciliary epithelium, glands, and goblet cells (17, 18). Moreover, both allow the passage of air into and out of the lungs and during inspiration the air is humidified, tempered, filtered, and supplied with nitric oxide before entering the lower airways. Nasal airconditioning capacities and filter function protect the lower airways from potentially harmful agents and it also participates to innate and adaptive immune defense (16). Patients with AR have partial or complete loss of function of the nose due to mucosal congestion and in this case the inhalation of cold or dry air can favor bronchoconstriction (19). Moreover, the nose protects the lower tract from inhaled foreign substances by filtering out the particles of 5–10 μm diameter, while the irritant and soluble gases are extensively removed by dissolution in nasal secretions (19). The lower airway acts similarly: the smaller inhaled particles are trapped and cleared by the mucociliary escalator (19). Furthermore, in case of disease, they share macroscopic pathological characteristics (Figure 1) and also the histological appearance is similar, with a comparable allergic response in rhinitis and asthma (Figure 2). The main difference between the nose and the lungs is that upper airway obstruction is mainly caused by vasodilatation and edema, whereas the lower airway patency is influenced by smooth muscle function.

## Epidemiologic Evidence

Asthma and rhinitis are a major public health problem because of their frequency, their impact on quality of life, school performance, and economic burden (1). The influence on patients’ quality of life has also been highlighted by a recent study on 2,896 children enrolled in the Taiwan Children Health Study, which demonstrated an association between allergic diseases, such as asthma and AR, and deficit hyperactivity disorder and oppositional defiant disorder (20). According to the International Study on Asthma and Allergy in Childhood, the prevalence of asthma worldwide was found to be 20%, and the prevalence of AR in Europe was observed to be 25% (1). Moreover, the prevalence of asthma and rhinitis varies all over the world: in countries with a more rural tradition, the numbers are usually lower than in countries with a higher level of urbanization (15, 19). Interestingly, the prevalence of asthma in subjects without rhinitis is usually less than 2% (17). The prevalence of AR appears to be at least triple the prevalence of asthma, and it has also been demonstrated that 19–38% of patients with AR have concomitant asthma and 30–80% of asthmatics have AR (15, 19).

## Pathophysiologic Evidence

### Allergic UAD

An immune response to external antigens induces the production of antibodies that are typically, but not exclusively, IgE antibodies. Allergic airway disease is caused by hypersensitivity or IgE-mediated reactions when inhaled allergen reacts with mast cells and basophils, the major effector cells, bearing IgE antibodies. The following cross-linking of allergen-specific IgE molecules bound to cells by allergen particles, results in the release of granule-associated mediators (i.e., histamine, tryptase), membrane lipid-derived mediators (i.e., leukotrienes), and cytokines (15, 20). Over time, the role of lipids in the pathogenesis of allergic disease has continued to expand thanks to sophisticated techniques capable of identifying and quantifying diverse lipid mediators (21). The early allergic response (maximal at 10–20 min) is usually characterized by edema, itching in the skin, rhinorrhea, sneezing, and erythema in the upper airways and bronchospasm, edema, mucous secretion, and cough in the lower respiratory tract. Instead, the late allergic response (within 2–6 h) in both the upper and lower airways is associated with an eosinophil activation and CD4 T cell tissue infiltrate, essential to maintain the chronic inflammatory process and tissue damage (20, 22).

To explain the interaction between the upper and lower airways, several mechanisms have been suggested (1, 16). The most likely mechanism is that localized inflammatory changes in the upper and lower airways leads to a systemic response, with bone marrow involvement, resulting in the release of progenitor cells that are then recruited to tissue sites. Inflammatory secretions may propagate from the upper airways to the lower *via* postnasal drip and systemic circulation. Although the nose is usually the first site of exposure to allergens or other noxious substances, despite a minimal nasal epithelial damage, a marked bronchial epithelial disruption may be present. Hence, researches hypothesized that the nasal mucosa has developed protective mechanisms that minimize remodeling and enhance epithelial regeneration (23). An important proof of the UAD interplay is the presence of epithelial basement membrane thickening, the typical hallmark of lower airway remodeling, not only in asthmatic patients but also in atopic patients without asthma and patients with AR. However, remodeling appears to be less extensive in the nasal mucosa than in the bronchial mucosa. The reason of this difference could be explained by the specific cytokine production of smooth muscle cells and by the presence of the genes of the embryologic differentiation in the nose and lungs or their different re-expression in asthma and rhinitis (24).

### Non-Allergic UAD

In contrast to allergic UAD, the etiology and mechanisms involved in non-allergic UAD is still unknown. In non-allergic asthma, it has been highlighted the importance of the presence of IgE in the bronchial mucosa, as in the nasal mucosa in LAR (10). However, it is still not clear what is the role of allergens in the asthmatic symptoms in patients with LAR. Some of the possibilities include allergy triggered by unknown antigens (fungi), persistent infection (caused by *Chlamydia trachomatis, Mycoplasma* spp., or viruses), and autoimmunity (10).

## Risk Factors

### Allergen Exposure

The allergen exposure has been considered the major risk factor for the development of UAD (15, 18). Rhinitis and asthma are characterized by a high prevalence of sensitization to those allergens that are common in the community, i.e., aeroallergens. When considering different aero/inhaled allergens, the most important distinction is that between outdoor allergens (i.e., pollen and molds), indoor allergens (i.e., cat, dog, mite), and occupational agents. Nevertheless, there are still important questions about the relevance of current allergen exposure to these diseases and to their management. Different factors can influence the IgE antibody response including genetics, allergen dose, and early life exposures that may inhibit or enhance the response (25).

### Genetic Factors

It is well known that allergic diseases run in families, implying a role for genetic factors in determining individual susceptibility: hereditability varies from 35 to 95% for asthma and from 33 to 91% for AR (26). Studies on the prevalence of allergic traits in relation to family history demonstrated incremental increases in risk of developing asthma or AR with the presence of one or both parents with allergic disease, and greater than three times the risk if allergic disease occurred in more than one first degree relative (26). To date, a positive family history remains one of the most reliable tools for prognosis of allergic disease (27). Moreover, the Multicentre Allergy Study has demonstrated that having parents with allergies is not only a strong predictor to develop any allergy but also strongly increases the risk of developing allergic multi-morbidity (28). A link of asthma and AR with different chromosomal regions was recently found thanks to genome-wide association studies. Actually, there are about 161 different potential biomarkers involved in respiratory inflammation (26). For example, Liu et al. have recently shown that the single-nucleotide polymorphisms in the TNFSF4 and FAM167A-BLK genes may be involved in asthma and AR gene (29) and Zhao et al. associated the PBX2 gene in the 6p21.3 asthma susceptibility locus with an increased risk for both AR and asthma (30). The genetic studies of allergic disease pave the way for tailored treatments to specific genotypes to improve therapeutic outcomes and minimize side effects.

### Other Risk Factors

Environmental exposures during pregnancy including diet, nutrient intake (especially vitamin D) (31), toxins (smoking, air pollution, microbes, infection) can alter the epigenome and interact with inherited genetic and epigenetic risk factors to directly and indirectly influence organ development and immune programming (32). Considering these data, the primary prevention of allergic disease should begin very early in life, even *in utero* (25).

## Emerging Biomarkers

### The Role of Microbiome

Microbiome is the totality of microbes, their genes, and their interactions in a given environment. It is increasingly accepted that human microbiome may play an important role by promoting the maturation of the host immune system. Thanks to advances in sequencing technologies, such as real-time quantitative PCR, it is now known that the microbes that inhabit healthy and diseased nose and lungs are different (33). According to the “hygiene hypothesis,” microbial exposures in early childhood may prevent allergies and asthma by modulating the Th1/Th2 and Treg imbalance (33). As matter of fact, children being raised on traditional farms have a much lower prevalence of allergic disease as children grown up in urban settings. The diversity of the microbial exposure has been shown to account for the asthma-protective farm effect. Nevertheless, in urban areas high exposure to environmental microbes also relates to a lower prevalence of allergic disease (15, 18). So, the microbiome itself could be considered as a potential biomarker source. A recent study demonstrated an association of asthma with reduced α- and β-diversity of the nasal microbiota and the relative abundance of a bacteria belonging to the *genus Moraxella*. The linking of asthma and *Moraxella*, however, was restricted to children not living on farms. In contrast to the nasal samples, the throat microbiota characteristics were not related to asthma (34).

### The Role of microRNA (miRNA)

microRNAs, a recently discovered regulators of gene expression, might be another non-invasive biomarkers to diagnose and characterize asthma and allergic. Circulating miRNAs have been considered to be involved in many inflammatory diseases, although gene regulation in the common inflammatory processes in UAD remains unclear (35). Panganiban et al. identified 30 miRNAs that were differentially expressed among healthy, allergic, and asthmatic subjects. These miRNAs fit into five different expression pattern groups. Among asthmatic patients, miRNA expression profiles identified two subtypes that differed by high or low peripheral eosinophil levels. Circulating miR-125b, miR-16, miR-299-5p, miR-126, miR-206, and miR-133b levels were most predictive of allergic and asthmatic status (35). These findings have shown the presence of the same particular miRNAs in different pathogenetic mechanisms of both AR and asthma, such as IL-13 pathway, GATA-binding protein 3, and mucin secretion. Interestingly, recent studies have shown that miRNAs could be used as potential pharmaceutical targets for anti-inflammatory treatment (36).

## Clinical and Treatment Evidence

The interaction between nose and lung in allergic airways disease is a bidirectional process, indeed it has been proved that the treatment of AR can improve asthma symptoms (15, 18). Subsequent ARIA updates and other reviews have made an attempt to summarize the diagnostic and therapeutic implications of this link based on these published evidence, but the evidence is still far from conclusive, due to limited number of randomized controlled trials available on subjects with concomitant AR and asthma (15, 18). Therapy for UAD is based on avoidance of the main allergens and irritants and pharmacotherapy [nasal and inhaled steroids, antihistamines, leukotriene receptor antagonists (LTRA), anti-IgE therapy, and allergen immunotherapy (AIT)] (11).

### Allergen Avoidance

Once allergy testing is complete, the physician may devise a comprehensive program of allergen avoidance. The lack of hay fever outside the pollen season indicates that complete allergen avoidance can be effective. Unfortunately, complete avoidance is rarely possible, especially for outdoor allergens. The effects of environmental control strategies have been most heavily studied with regard to dust mites and furry pets (37). Compliance with these measures may be difficult but will certainly be helpful in many patients with hypersensitivity to these allergens. Avoidance of other rhinitis and asthma triggers, such as cigarette smoke, outdoor pollutants, fumes, and irritants, is sensible in clinical practice (38).

### Pharmacologic Therapy

Oral and/or intranasal antihistamines and nasal corticosteroids are both appropriate for first-line AR treatment although the latter are more effective (39, 40).

Some authors reported a decrease in asthma symptoms and AR after intranasal corticosteroid treatment of rhinitis and a recent meta-analysis confirmed the beneficial effect of intranasal steroids in AR (41). LTRAs are known to be useful for long-term management of asthma patients complicated by AR (42). Leukotrienes are generated by the metabolism of arachidonic acid *via* the 5-lipoxygenase (5-LO) pathway, which is involved in the rapid initial inflammation response. LTRAs block the cysteinyl-leukotriene receptor, which are peptide-conjugated lipids produced by activated basophils, eosinophils, mast cells, and macrophages, to relieve the symptom of AR (42). Recent studies demonstrated that LTRAs have a significant influence in improving patients’ nasal symptoms and quality of life and the use of LTRAs in combination with antihistamines has generally resulted in greater efficacy than when these agents were used alone (43). The recombinant, humanized, monoclonal anti-IgE antibody (Omalizumab) forms complexes with free IgE, blocking its interaction with mast cells and basophils and lowering free IgE levels in the circulation (44). Omalizumab has been tested in several clinical trials, and its beneficial effect has been established in patients with uncontrolled allergic asthma, leading to its approval by FDA (38). In patients with severe asthma and rhinitis, omalizumab improved nasal and bronchial symptoms and reduced unscheduled visits due to asthma. The clinical benefit of treatment with omalizumab is associated with an anti-inflammatory effect on cellular markers in blood and nasal tissue, as well as with a reduction in FcRI expression and function (45).

The only treatment potentially able to interfere with the natural history of respiratory allergy is AIT, specifically aimed at modifying the response to sensitizing allergens (37). Current data support the effectiveness of AIT in AR and a beneficial effect in allergic asthma (38). In particular, AIT may prevent the onset of asthma by halting the progression from rhinitis, by preventing new sensitizations or by avoiding the primary development of allergy (46). In the context of the UAD, AIT consistently shows a clear benefit for both the upper and the lower airways.

## Conclusion

The link between upper and lower airways, the so-called UAD, has been revealed by several epidemiologic, pathophysiologic, and clinical evidences, changing the global pathogenic view of respiratory allergy. AR and asthma are both manifestations of a single inflammatory process and require an integrated diagnostic and therapeutic approach in order to get global disease control.

## Author Contributions

All authors made substantial contribution to the conception of the work, reviewed the literature on the subject, and drafted the final version of the manuscript; AL and GM revised it critically for important intellectual content. All authors finally approved the version to be published and agreed to be accountable for all aspects of the work in ensuring that questions related to the accuracy or integrity of any part of the work are appropriately investigated and resolved.

## Disclaimer

The authors declare that written informed consent was obtained by the patients for the use of the images.

## Conflict of Interest Statement

The authors declare that the research was conducted in the absence of any commercial or financial relationships that could be construed as a potential conflict of interest.
